# The metasomatic enrichment of Li in psammopelitic units at San José-Valdeflórez, Central Iberian Zone, Spain: a new type of lithium deposit

**DOI:** 10.1038/s41598-020-67520-6

**Published:** 2020-07-02

**Authors:** Alfonso Pesquera, Encarnación Roda-Robles, Pedro P. Gil-Crespo, David Valls, José Torres Ruiz

**Affiliations:** 1 Dpto. de Mineralogía Y Petrología, Universidad del País Vasco (UPV/EHU), Campus de Bizkaia, Sarriena s/n, 48940 Leioa, Bizkaia Spain; 2 TECNOLOGÍA EXTREMEÑA DEL LITIO, S.L., C/ Juan de La Cierva, 18. Polígono Industrial Mejostilla, 10004 Cáceres, Spain; 3 Dpto. de Mineralogía Y Petrología, Universidad de Granada, Fuentenueva s/n, 18071 Granada, Spain

**Keywords:** Solid Earth sciences, Energy science and technology

## Abstract

A new type of Li mineralisation in hard rock has been found to occur in the Valdeflórez area (Cáceres, Spain), where there is 111.3 Mt of resources and a mean value of 0.61 wt% of Li_2_O. Lithium is mainly held by very fine-grained micas, important constituents of Ordovician psammopelitic rocks belonging to the Palaeozoic metasedimentary sequence of the Cáceres syncline. The mineralised zone has an elliptical surface shape with a dimension of ~ 700 × 500 m. Lithium-bearing rocks show a characteristic layered appearance, in which light grey quartz-micaceous laminae < 1 mm to some centimetres in thickness, with a variable ratio of quartz to mica, alternate with fine to very fine-grained, dark grey to black tourmalinite laminae parallel to the regional foliation. Subvertical quartz + (montebrasite)-veins that cut the regional foliation at an extremely high angle are also common in this area. Mineralisation and the associated veins are likely to be linked to the intrusion of the nearby Cabeza de Araya pluton. The infiltration of granite-derived Li-, F-, B- and P-rich aqueous fluids into the host rocks through fractures related to shearing processes is considered to be the cause of the formation of Li-rich micas and intense tourmalinisation at the expense of pre-existing phyllosilicates.

## Introduction

Discovered in 1817 by the Swedish chemist Arfwedson in petalite, lithium is an important rare element; it is known as the energy metal and is promoted worldwide. The demand for lithium has increased in recent decades because of its application as a raw material for ceramic and glass, as rechargeable batteries of electric vehicles, mobile phones, laptops and digital cameras and for light aircrafts, high-speed trains alloys and nuclear fusion fuel. Lithium is also used in some nonrechargeable batteries for heart pacemakers, for treating some mental illnesses and for toys and clocks^1^.

Lithium concentrations in the Earth’s upper crust is 24 ppm^2^. In igneous rocks, the abundance is typically 28–30 ppm, but in sedimentary rocks, it can be as high as 53–60 ppm^3,4^. Mines and salt lakes generally are believed to contain a total of 14 × 10^6^ tonnes of Li. The Li concentration in seawater is quite low (0.1–0.20 ppm), but the total amount of Li is estimated to be ≈ 230 × 10^9^ tons^5^. This becomes an attractive source for this element, leading to an increasing investigation into the separation and recovery of Li from seawater^5–8^. However, the low concentration of lithium in seawater requires the processing of large volumes of water to extract even small quantities of the metal. In addition, seawater contains a variety of dissolved minerals, many of which are present in much greater quantities than lithium. As a result, it is exceedingly difficult or nearly impossible for traditional separation technologies, such as membrane filtration, ion exchange and reverse osmosis, to extract lithium from seawater without excessive energy consumption or the fouling of filtration media and/or chemical regenerants.

Lithium is sourced mainly through salt lake brines and pegmatites. Until the 1990s, the lithium chemical and metal market was dominated by American production from mineral deposits, but by the turn of the twentieth century, most production came from non-U.S. sources. The largest reserves of Li are found in evaporitic-type deposits, particularly in Bolivia, Chile and Argentina, with ≈ 56% of the world’s reserves occurring in these countries^3^. In addition to these types of mineralisation, lithium-bearing clay deposits have been estimated to contain about 7% of the world’s lithium resources^3^.

Rare-element granitic pegmatites may also have concentrations that are high enough to make their mining economically profitable. A significant number of pegmatites are being mined or explored for Li^9,10^. Spodumene, lepidolite, petalite, eucryptite and amblygonite-montebrasite are the most common Li-bearing minerals found in economic deposits of this type, with spodumene being the most important. Lithium-enriched rocks are common in the Central Iberian Zone (CIZ), mainly in aplite-pegmatite bodies grouped in pegmatite fields, which are usually spatially related to larger granite intrusions^11,12^. Less commonly, Li occurs in concentrated forms in quartz-rich and greisen-type veins that constitute stockworks at the top of more or less fractionated leucogranitic cupolas. In these veins, the main Li-carrier minerals belong to the amblygonite-montebrasite series^11,12^.

Lithium mineralisation in the Valdeflórez area (Spain) represents a new mode of occurrence of large-scale Li enrichment in metamorphic rocks, which nowadays constitutes the San José-Valdeflórez mining project. This is an important mineralisation of Li in metasedimentary rocks that was presumably induced by granite-derived Li- and B-rich fluids. Here, we focus on the geological characteristics that distinguish this from other deposits of Li, the mode of occurrence and the composition of the lithium-rich minerals, spatial distribution of lithium in the area and the possible genetic model. In this way, we propose a new type of Li deposit in hard rock.

## Geological setting

**Figure 1:**
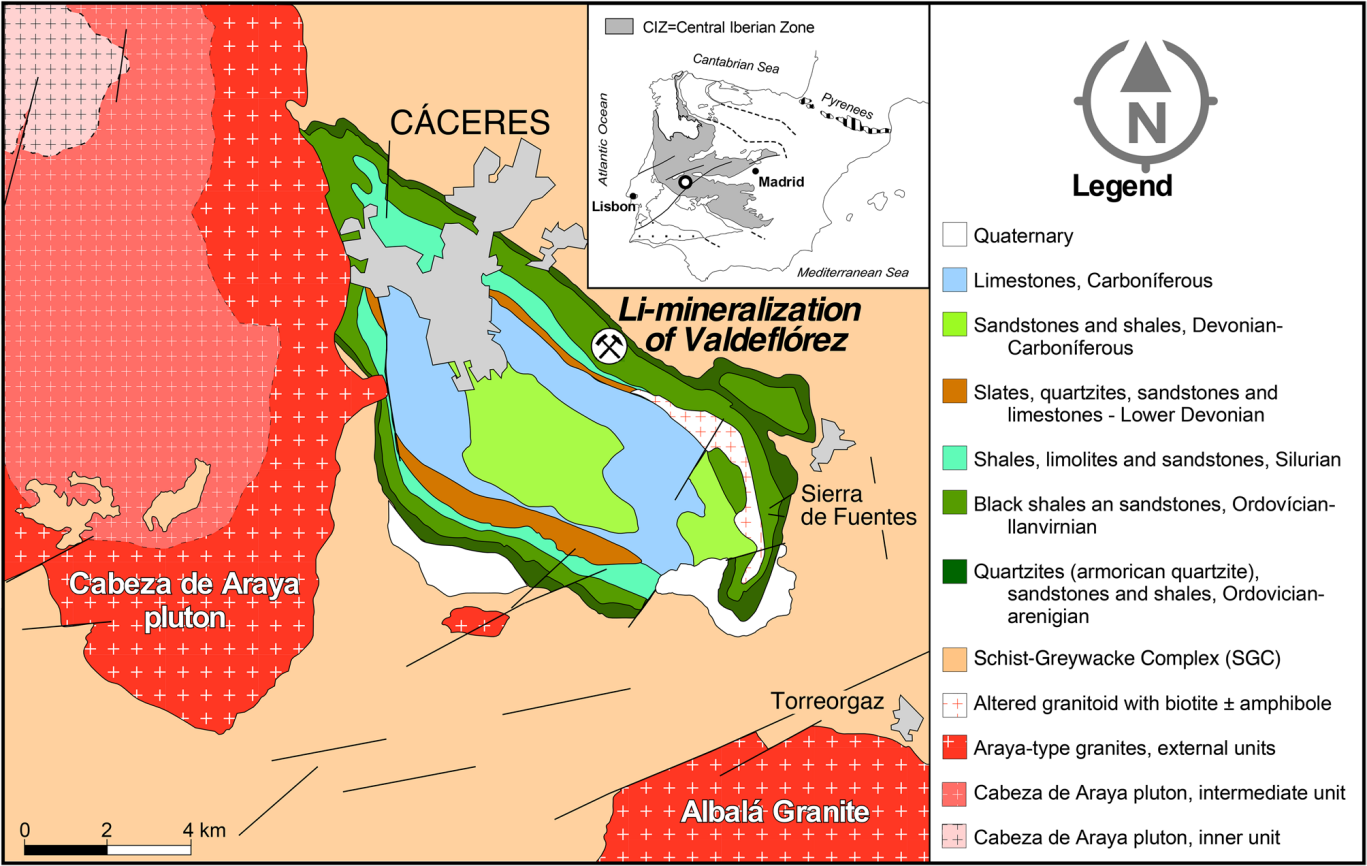
Simplified geological setting of the Valdeflórez area (modified from Palacios, Eguiluz, Apalategui, Jensen, Martínez-Torres, Carracedo, Gil-Ibarguchi, Sarrionaindia and Martí ^56^and Campos-Egea^51^). This map has been generated by using the software Canvas Draw v.5 (CANVAS, https://www.canvasgfx.com/en/products/canvas-x-draw/).

The Valdeflórez area is located in the southern part of the CIZ, in the province of Cáceres, Spain (Fig. 1). The CIZ constitutes the innermost region of the Iberian Variscan belt and represents the westernmost segment of the European Variscides. Two main domains can be distinguished in the CIZ: (i) the northern CIZ, which mainly consists of a thick Neoproterozoic to Early Palaeozoic metasedimentary sequence that is intruded by a set of Cambro-Ordovician metagranites, and (ii) the southern CIZ, which includes a very thick sequence of Neoproterozoic to Lower Cambrian psammopelitic metasediments with some conglomeratic, carbonate and volcaniclastic rock intercalations, the so-called Schist-Greywacke Complex. In some areas, early Ordovician to early Carboniferous sediments occur unconformably over these metasedimentary sequences. The northern and southern CIZ domains show differences in the tectonic style, geochemical features and metamorphic grade^13,14^.

The most prominent structural features of the CIZ were acquired during the Variscan orogeny, which caused a polyphasic deformation (see^15^ for an overview). The main phases, D1 and D2, yielded a heterogeneous ductile deformation with a regional low- to high-grade metamorphism that evolved from intermediate-P/high-T to low-P/high-T conditions, reaching partial melting in the lower structural levels^13,16,17^. Subsequent phases of deformation developed under low-grade metamorphic conditions, which are considered as responsible for CIZ’s macrostructural models and retrograde effects on previous metamorphic mineral associations.

The CIZ is characterised by a huge volume of syn- to postkynematic granitoids that were emplaced between ~ 330 and ~ 290 Ma^18–20^, where four S-types (S1, S2, S3 and S4) and one I-type granite series have been documented^21^. Peraluminous granitoids (A/CNK ratios ≥ 1.1) with a crustal isotopic signature (^87^Sr/^86^Sr ~ 0.711–0.715 and εNd ~  − 4 to − 8^18^) constitute an important rock suite, but metaluminous granitoids and basic rocks are scarce in the CIZ^22–24^.

Many ore deposits are genetically related to granitic massifs in this geologic domain, particularly in the western part, including pegmatites, aplites, stockworks and quartz- and greisen-type veins. Although most of the pegmatites exhibit a poor degree of evolution, Li is commonly enriched in some fractionated pegmatitic bodies (usually aplite-pegmatites) and in some quartz-rich veins^11,12^. According to field relationships and geochemical affinities, the S1 and S2 granitic series—interpreted as deriving mainly from the partial melting of highly peraluminous, Ca-poor and P-rich Neoproterozoic metasediments during the Variscan orogeny—are related to Li mineralisation in the CIZ^12^.

## Methods

Electron-microprobe analyses (640) of the micas and tourmaline were obtained using a CAMECA SX100 at the research centre of the University of Oviedo (Spain). The operating conditions included a voltage of 15 kV, a beam current of 30 nA and a beam diameter of about 2 μm. The standards for calibration were as follows: orthoclase (K and Si), wollastonite (Ca), MnTi (Mn and Ti), magnetite (Fe), albite (Na), corundum (Al), forsterite (Mg), Cs glass (Cs), apatite (P), IRX (Rb) and LiF (F). Data were reduced according to the procedure by Pouchou and Pichoir^25^. Analytical errors are in the ranges of ± 1–2% (major elements) and ± 10% (minor elements). Structural formulae for the micas were established based on 22 atoms of oxygens, and tourmaline formulae were normalised based on 15 cations exclusive of Na, Ca and K, according to the scheme by Henry and Dutrow^26^.

Trace elements, including Rb, Cs, Sn, Zn, Ba, Be, Nb, Ta, Zr, Ni, Tl, Pb, P, Sc, V, Ni, Ga, Sr, Y, Zr, Mo, Hf, W, Tl, Th, U and REE, were analysed by Quadropolar Laser-Ablation Inductively Coupled Plasma Mass Spectrometry (LA-Q-ICP-MS) (iCAP-Qc model) in SGIker at the Universidad del País Vasco (UPV/EHU) (see^27^). The LA-Q-ICP-MS analyses were conducted with a 213 nm New Wave Research UP213 laser coupled with a Thermo Fisher Xseries-II ICP-MS with an Xt interface and a shielded plasma torch using the NIST SRM 612 glass for tuning. The ablation was carried out in a helium atmosphere. The laser beam was fixed to an 80-μm-wide square section. The spot was preablated for 45 s using a laser repetition rate of 10 Hz and 40% output energy. Then, the spot was ablated for 60 s at 10 Hz with a laser output energy of 75%. A typical session of analysis of a single thin section began and ended with an analysis of the calibration standard NIST-612 glass, which was also analysed every five spots to correct for drift. Silicon was used as the internal standard, and the accuracy and precision were based on the measurement of secondary standards (NIST-610). The limits of detection (LOD) were based on three times the standard deviation (3σ) of the measurements. Data reduction was carried out with Iolite 3 software^28^ (https://iolite-software.com). Taking into account the good positive correlation between the Li and F contents in the micas, which is consistent with the empirical relationships offered by Henderson, Martin and Mason^29^, Tindle and Webb^30^, and Tischendorf, Gottesmann, Förster and Trumbull^31^, the Li_2_O values in trioctahedral micas were estimated from microprobe data using the equation tri-4b of^29^, whereas those of the dioctahedral micas have been calculated using the di-1 equation of the same authors.

It is necessary to mention the specific problem of obtaining good chemical data on samples that are mostly very fine- to ultrafine-grained. To overcome this challenge, only those chemical analyses (≈ 80%) that fit sufficiently with the ideal formula have been considered. The others probably do not correspond to analyses of a single crystal, but to a mixture of fine-grained crystals of different minerals.

## Geology and mineralogy of the host rocks and Li mineralisation

The Valdeflórez area is located on the northern flank of the Cáceres syncline, near the Cabeza de Araya pluton (Fig. 1). This pluton is the most representative of the granitoids of the so-called “serie mixta”^22^ and belongs to the S2 granitic series in the classification of Villaseca^21^. It consists of three main units with an emplacement sequence from 308 to 305 Ma^30^ (Fig. 1): (i) cordierite-bearing two-mica granite with feldspar megacrysts and biotite >  > muscovite in the outer zone; (ii) a coarse-grained two-mica granite in the intermediate zone; and (iii) aplitic granite and leucogranite in the inner zone, with a mineral assemblage similar to (ii) but with albite and a higher proportion of muscovite^31^. Andalusite, sillimanite, tourmaline and cordierite are usually among the accessory minerals in these granites. Granites of the outer and intermediate zones appear to be related by a fractional crystallisation process, as illustrated by their geochemical trends^31^. In addition, some two-mica granites display relatively high Na, Rb and Li contents that can be attributed to enrichment by late-magmatic fluids. In contrast, the granites in the inner zone do not seem to form part of the differentiation series defined by the other granites, as evidenced by their discontinuous geochemical trend^31^.

**Figure 2:**
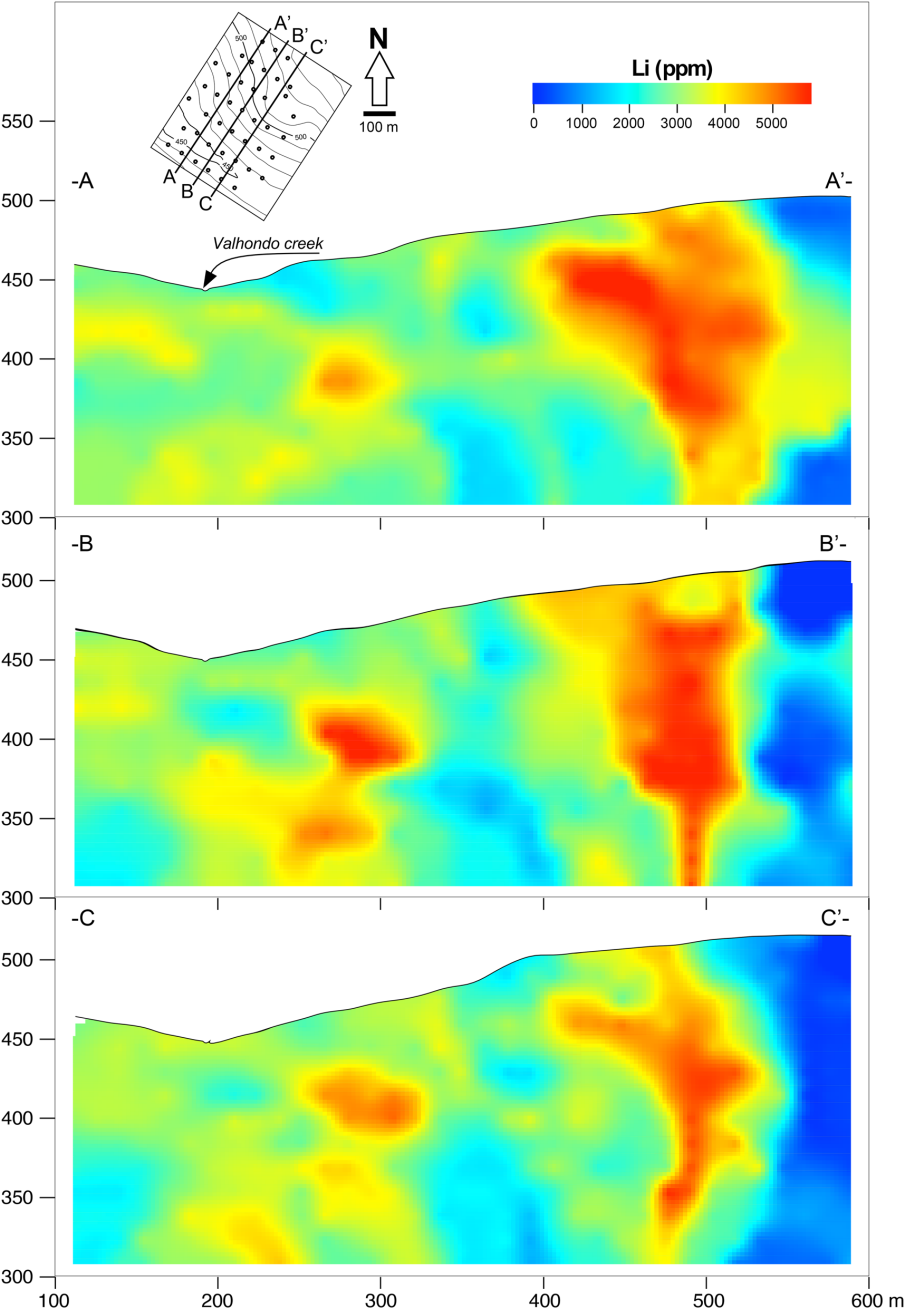
Cross-section showing the Li contents in the Valdeflórez area. These figures have been generated by using the software Aabel v.3 (GIGAWIZ, https://www.gigawiz.com/) and the software Canvas Draw v.5 (CANVAS, https://www.canvasgfx.com/en/products/canvas-x-draw/).

The Li mineralisation consists of metasomatised metasedimentary units, including a psammopelitic sequence with some interlayered quartzitic levels (Fig. 1). Metapelites are fine-grained (< 100 μm) and, in general, display a spaced to continuous foliation on a thin section scale defined by the micas. Post-S1 partially chloritised biotite, which developed during contact metamorphism, appears at some locations. Metapsammites and quartzites show a massive aspect with a granoblastic texture, in which variable proportions of quartz and micas and minor feldspar, constitute the main mineral association. Tourmaline, zircon, rutile and ilmenite occur as accessory phases. The mineralised zone has an elliptical surface shape with a dimension of ~ 700 × 500 m (Fig. 2). A drilling programme revealed that mineralisation extends at least 450 m in depth, dipping subvertically to the north west; it largely inherits the primary fabric and is characterised by the occurrence of a repetitive lithological sequence of a millimetre to centimetre thickness, where the following alternate: (i) very fine- to fine-grained, light grey predominantly micaceous layers; (ii) very fine- to fine-grained, dark grey to black tourmalinite; and (iii) layers with intermediate compositions in which quartz, micas and tourmaline show variable proportions. Quartz- and greisen-type veins that strike mainly NE-SW, dip subvertically to the NW and cut the regional foliation at a very high angle are also common in this area. Vein thicknesses vary from around 1 cm to a few metres in places, with average values of 2–5 cm. Locally, the veins branch and cross-cut the host rock parallel to and across the regional foliation, in places forming stockworks. No clear evidence of compositional gradients with proximity to the veins has been observed.

### Evaluation of the Li mineralisation of San José-Valdeflórez

Based on the study made by Infinity Lithium^32^, with ≈ 10,500 m of drilling and more than 4,200 chemical analyses, the San José-Valdeflórez mineralization can count on 111.2 Mt of resources (indicated + inferred), with a mean value of 0.61 wt% of Li_2_O (Table 1). This means that the Li deposit of Valdeflores would be the second largest one in Europe and the first to be mined in an open pit. Representative cross-sections of the mineralised body, with the distribution of Li contents, are illustrated in Fig. 2.

**Table 1:** Evaluated resources for the San José-Valdeflórez Li-mineralization.

	Resources (Mt)	Li (%)	Li_2_O (%)	Sn (ppm)
Indicated	59.0	0.29	0.63	217
Inferred	52.2	0.27	0.59	193
Total	111.2	0.28	0.61	206

### Li-rich quartz-micas rocks

**Figure 3:**
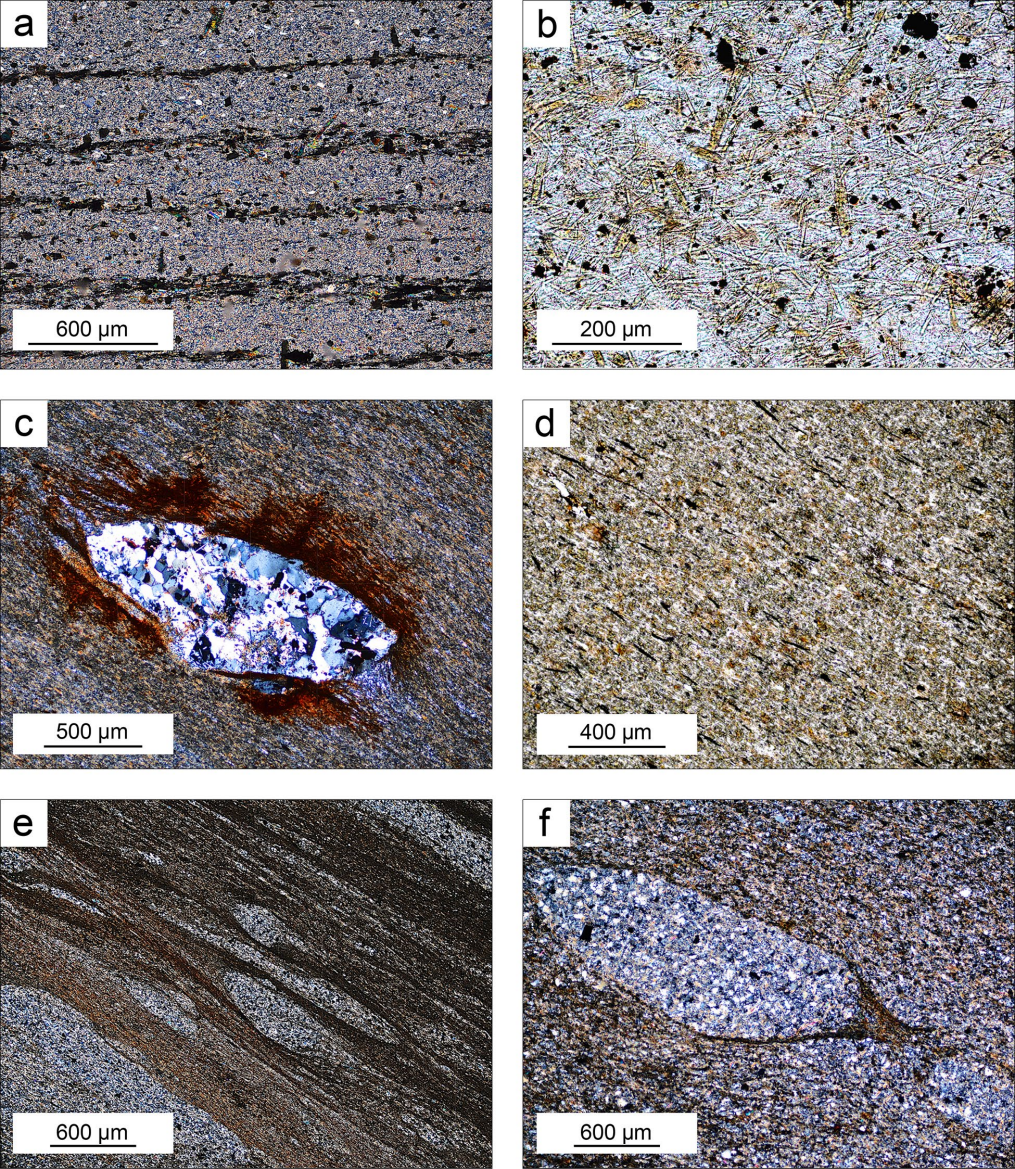
(**a**) Very fine-grained quartz-mica layer containing tourmaline-rich laminae. (**b**) Fine-grained tourmaline network filled with ultrafine-grained Li-rich micas; (**c**) white mica flakes inside quartz polycrystalline augen in tourmaline-rich rock; (**d**) fine-grained tourmalinite with a foliation mainly defined by the micas. (**e**) Very fine-grained tourmalinite displaying psammitic lenses and σ-type porphyroclasts; (**f**) microboudinage of psammitic augen in tourmaline-rich rock. Notice the beard-like aggregate of tourmaline and carbonaceous matter between the separate boudins. (Pictures obtained by the authors).

Overall, the quartz-micaceous layers consist of micas, quartz in variable proportions, minor tourmaline and apatite, zircon, ilmenite, rutile, pyrite and arsenopyrite as accessory minerals. White mica predominates over dark mica, and they commonly are intimately associated at a microscopic scale. In these rocks, the micas display a variety of sizes, colours and textures^33^ but mostly occur (i) as compact layers and laminae with variable amounts of disseminated very fine-grained quartz clasts (Fig. 3a) and (ii) as filling the fine-grained tourmaline network in tourmalinite (Fig. 3b). White micas locally exhibit yellowish, beige or pinkish hues. Unlike the metamorphic reddish-brown biotite, the dark mica of the metasomatic zone is optically heterogeneous with a variable colour from pale grey or orange to pinkish brown. White mica flakes (< 200 μm) inside quartz-rich augen containing opaque minerals can also be observed (Fig. 3c).

**Figure 4:**
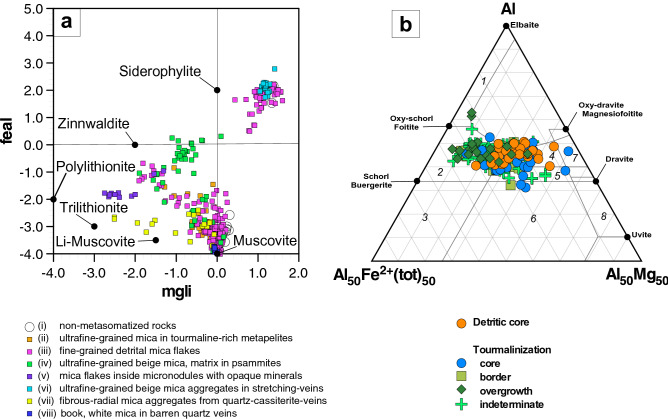
(**a**) Mica compositions from Valdeflórez area in the diagram of Tischendorf et al.^29^ [(mgli = Mg–Li (apfu), feal = Fetot + Mn + Ti–VIAl (apfu)]. (**b**) Al–Fe(tot)–Mg ternary diagram (in atomic proportions) for tourmalines of the Valdeflórez area. Numbered fields after Henry and Guidotti^57^: (i) Li-rich granitoids, pegmatites and aplites, (ii) Li-poor granitoids, pegmatites and aplites, (iii) hydrothermally altered granitic rocks, (iv) metapelites and metapsammites (aluminous), (v) metapelites and metapsammites (Al-poor), (vi) Fe^3^^+^-rich quartz–tourmaline rocks, calc-silicates and metapelites, (vii) low-Ca meta-ultramafics, and (viii) metacarbonates.^57^.

Representative microprobe analyses and LA-ICP data show significant compositional variations in SiO_2_ (45–53 wt%), Al_2_O_3_ (20.5–36.4 wt%) FeO (0.4–22.7 wt%), MgO (0.1–6.2 wt%), K_2_O (5.75–9.8 wt%), Li_2_O (up to 6.0 wt%), Cs_2_O (up to 2.8 wt%), Rb_2_O (up to 0.5 wt%) and F (up to 8.0 wt%). A representation of the data on a (Fe + Mn + Ti–^VI^Al)/(Mg–Li) diagram29 shows compositional trends from muscovite to trilithionite, muscovite to zinnwaldite and biotite to zinnwaldite (Fig. 4a). The higher FeO contents correspond to reddish-brown micas, which approach typical biotite compositions and have relatively low F and Cs contents. The fluorine intercept values for biotites, here by using the method described by Munoz and Ludington^34^, are in the range of 1.25–1.46 with an average of 1.31 ± 0.07.

### Tourmaline-rich rocks

These rocks are very fine- to fine-grained, dark green to black and contrast sharply with the light-coloured quartz-mica rocks. A characteristic feature is a layered appearance where tourmaline-rich laminae < 1 mm to some centimetres in thickness alternate with quartz-mica laminae (Fig. 3a,d,e and see Fig. 3a,b in^35^). The percentage of tourmaline is variable, from < 20% to ≈ 80% by volume, with subordinate white micas, quartz and ore minerals. In the thin sections, tourmaline appears as pale green-colourless to dark green or yellowish brown, euhedral to subhedral crystals (< 50 ~ μm generally), without a distinctive lineation, forming a more or less dense network filled by a fine-grained quartz-micaceous matrix (Fig. 3b). However, mylonitic textures with very fine-grained psammitic lenses, σ-type psammitic to quartzitic porpyroclasts and boudinage are also common (Fig. 3e,f).

Microprobe analyses of the Valdeflórez tourmaline show substantial compositional variations in FeO (5.02–14.69 wt%), MgO (0.97–8.20 wt%), and TiO_2_ (0.17–2.00 wt%). Smaller proportional variations occur for Al_2_O_3_ (28.77–34.73 wt%), SiO_2_ (32.79–39.21 wt%), CaO (0.02–2.40 wt%), Na_2_O (1.30–2.66 wt%) and F (0.01–1.67 wt%) (Fig. 4b). Amounts of MnO are very low (< 0.15 wt%), and Li contents (< 80 ppm) are negligible.

### Quartz- and greisen-type veins

A set of white mica-bearing quartz- and greisen-type veins with cassiterite and minor amounts of K-feldspar ± amblygonite-montebrasite ± apatite ± fluorite ± topaz traverses the mineralised area. Micas from veins are mostly prismatic muscovite with 0.4–0.8 wt% F and 0.1–0.3% wt% Li_2_O and very fine-grained Li micas with 0.1–7.8 wt% F and up to 5.1 wt% Li_2_O. A detailed petrographic and mineralogical description of these veins can be found in Pesquera, Torres-Ruiz, Gil-Crespo and Velilla^33^, and Torres-Ruiz, Pesquera, Gil and Casas^35^.

## Discussion

It is obvious that the micas of the psammopelitic metasediments are the main host for Li in the Valdeflórez area. Outside the mineralised zone, the micaceous minerals include muscovite and minor biotite. Petrographic observations, in conjunction with chemical and experimental data, indicate the following: (i) micas underwent a variable enrichment in Li and other elements as a result of the metasomatic interaction of granite-derived fluids with metasedimentary rocks; (ii) dark and white micas show convergent compositional trends with increasing Li, F and FeO contents; (iii) the Li metasomatism is concomitant with that of boron; and (iv) the general cation deficiency in the interlayer site of the micas reflects the physical–chemical conditions of the metasomatic processes developed in the Valdeflórez area. Based on the compositional trends of the micas (Fig. 4a), the formation of zinnwaldite and trilithionite from biotite and muscovite by reacting with Li- and F-rich fluids may have developed via the substitutions Si^IV^Al_0.5_^VI^Li_1.5_^VI^Al_−1_^IV^Fe_−2_, Fe^VI^Li^VI^Al_−1_^VI^ and Li_3_^VI^Al_−1_^VI^Xv_−2_, where Xv represents the vacancies in the octahedral sites. Likewise, the formation of tourmaline from biotite reaction with boron-bearing aqueous fluids may have derived from reactions of the following type:

$$6{\text{biotite}} + 3{\text{B}}_{2} {\text{O}}_{3} + 2{\text{Na}}^{ + } + 8{\text{H}}^{ + } + {\text{H}}_{2} {\text{O}} = 2{\text{tourmaline}} + 3{\text{SiO}}_{2} + 6{\text{K}}^{ + } + 9\left( {{\text{Fe}},{\text{Mg}}} \right)^{2 + } + 14{\text{OH}}^{ - }$$

Similar reactions have been inferred from fluid–rock interaction experiments between biotite-rich schist and boron-bearing aqueous fluids under different physical–chemical conditions^36^. In the case of Valdeflórez, Al, Fe and Mg are supplied by the metasedimentary rocks, while fluids would be provided by Li, F, B, P and, to a lesser extent, Rb and Cs. Temperature estimates with the Ti-in-biotite thermometer^37^ for biotites with an average of Mg/(Mg + Fe + Mn) = 0.32 ± 0.034 and Ti = 0.14 ± 0.03 apfu yield temperatures of 400–450 °C, which are considered the highest temperatures at which the metasomatic processes developed. Likewise, the use of the calibrations by Munoz and Ludington^34^ to calculate the activity ratio aH_2_O/aHF of aqueous fluids in equilibrium with biotite suggests that the log (fH_2_O/fHF) values in the fluid phase are around 4.4 for the temperature and biotite compositions indicated above. A high activity of F^-^ in the hydrothermal fluids may be of paramount importance for the transport of Li because Li^+^ and F^-^ (as well as PO_4_^3−^) are considered to be hard metal and ligands, respectively^38^, and tend to form strong complexes in hydrothermal fluids^39^.

Stockworks constituted by montebrasite ± cassiterite quartz veins, which are very similar to those of Valdeflórez, are observed in other localities of the CIZ and are related to fractionated granitic rocks in cupolas (e.g., Golpejas, Barquilla^40^; Massueime^41^; El Trasquilón^42^). The spatial distribution of these quartz veins and the extensive associated Li–B metasomatism in the Valdeflórez area suggest that Li enrichment, tourmalinisation and mineralised quartz- and greisen-type veins are closely related to fluids derived from the same granitic source in a tectonically active regime. Drilling works down to 450 m in depth have not found any granitic body under the mineralisation. However, there are several lines of evidence to envisage the Cabeza de Araya pluton as the source of Li- and B-rich fluids. (1) Li mineralisation is located near the pluton, with the occurrence of some Araya-type granitic apophysis in the Valdeflórez area (Fig. 1). In addition, gravimetric data reveal the pluton extends largely in depth to the south east^43^. (2) The Cabeza de Araya pluton belongs to the S2 granitic suite, which is related to Li mineralisations in the southern CIZ^12^. The pluton-forming units have relatively high Li contents (147, 212 and 175 Li ppm for the external, intermediate and internal granitic units, respectively^31^). (3) In the westernmost corner of the pluton (Segura, Portugal^44^), Li-rich aplite-pegmatites can be found.

**Figure 5:**
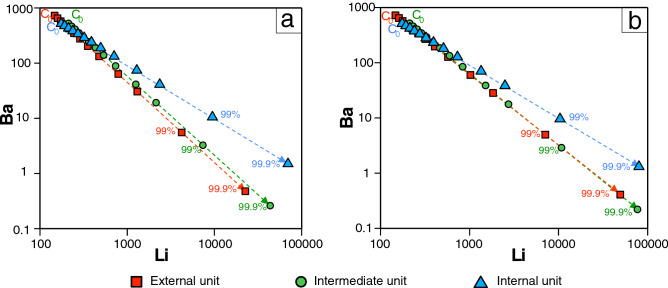
Plots of Li vs. Ba for the three granitic units of the Cabeza de Araya pluton (C_0_) and the possible paths of fractional crystallisation starting from the composition of C_0_ using (**a**) the most favourable distribution coefficients for Li fractionation and (**b**) the less favourable ones, per the given modal proportions. Distribution coefficients compiled by other authors^45, 46^.

To assess the potential of the different granitic units from the Cabeza de Araya pluton to fractionate and exsolve Li-rich fluids, a geochemical modelling using the Rayleigh equation for fractional crystallisation has been carried out (Fig. 5):

$${\text{C}}_{1} /{\text{C}}_{0} = {\text{F}}^{{({\text{D}} - 1)}}$$

where C_1_ is the weight concentration of a trace element in the differentiated melt, C_0_ is the weight concentration of that element in the parent melt, F is the fraction of melt, and D is the bulk distribution coefficient. Modelling has been done starting from the Li and Ba contents of the three main petrographic units described in the pluton (Fig. 1). Modal proportions of the minerals of these granitic units have been used as fractional crystallisation components. It should be noted that to apply this equation, it is needed to know the distribution coefficients of Li and Ba between solid and melt. Inasmuch as the aim of this modelling is just to check the potential of the three Cabeza de Araya pluton-constituting units to generate Li-rich melts and fluids, two different sets of distribution coefficients have been used: one being the most favourable for Li fractionation based on the given modal proportions (Fig. 5a) and the other one being the less favourable for it (Fig. 5b) (data compiled by Jolliff, Papike and Shearer ^45^ and London^46^). Degrees of fractionation of granitic melts up to 99% are proposed to obtain the concentrations of Li observed in the aplite-pegmatites of some fields and in some leucogranitic cupolas in the CIZ (e.g.,^47–49^). According to the results obtained for the Cabeza de Araya pluton, the concentration of Li in the fractionated melts would be in the order of thousands of ppm for the three granitic units. Accordingly, the fractional crystallisation of a melt with the composition of any of the three units of the Cabeza de Araya pluton can lead to significant Li concentrations, presumably high enough to exsolve Li–(B–F–P–Sn)-enriched hot fluids during a hydrothermal stage. Experimental work with hydrous granitic melts indicates that the liquidus and solidus may be lowered with the presence of B, Li, F and P, which also enhances the solubility of H_2_O and controls the behaviour of incompatible lithophile elements (e.g.,^50–53^).

The shearing processes related to D3 deformation may have triggered an abrupt change in the evolution of the system from closed to open. Fluorine is generally considered to be a compatible element in fluid-saturated melts^54^ and is not lost into the volatile phase until there are very low pressures^55^. Accordingly, a sharp drop in pressure when the system is suddenly opened through shear processes is expected to provoke the exsolution of the Li–F–B–P–rich fluids responsible for the Li mineralisation of Valdeflórez.

## Conclusions

The salient points of the present study are as follows:

1. An epigenetic origin for Li- and B-rich metasedimentary rocks related to the evolution of the Cabeza de Araya pluton appears to be the most plausible model based on the following: (i) the proximity of the pluton, which belongs to the S2 granitic series that are parental to the abundant Li mineralisation occurring in other localities of the southern CIZ; (ii) petrographic, mineralogical and geochemical characteristics; (iii) the low concentrations of B, Li, F, Cs, P and Sn in nonmetasomatised metasediments.
2. The extensive Li–B metasomatism and distribution of the associated veins in the Valdeflórez area suggest that Li-rich mica layers and tourmalinite formations are related to the activity of fluids derived from the same granitic source in a tectonically active environment.
3. The layered appearance of the Li mineralisation in which quartz-mica laminae alternate with tourmaline-rich laminae is the result of the following: (i) the primary fabric of the metasedimentary rock and (ii) the selective Li-B metasomatism along the psammopelitic layers that is controlled by the preferential incorporation of Li and Fe–Mg in micas and tourmaline, respectively.
4. The Li mineralisation of Valdeflórez can be considered a new type of Li deposit based on geological, mineralogical and geochemical data, as well as the size, concentration and reserves of the metasomatised zone.
